# Response of Summer Maize Photosynthate Accumulation and Distribution to Shading Stress Assessed by Using ^13^CO_2_ Stable Isotope Tracer in the Field

**DOI:** 10.3389/fpls.2017.01821

**Published:** 2017-10-26

**Authors:** Jia Gao, Bin Zhao, Shuting Dong, Peng Liu, Baizhao Ren, Jiwang Zhang

**Affiliations:** State Key Laboratory of Crop Biology and College of Agronomy, Shandong Agricultural University, Taian, China

**Keywords:** summer maize, photosynthetic characteristics, shading in the field, ^13^C photosynthate distribution, grain yield

## Abstract

Maize is one of the most important crops globally that provides food, feed, and bioenergy. However, shading stress threatens maize production. In this study, we investigated the effects of shading on photosynthate accumulation and distribution of summer maize in the field. Zhengdan958 (ZD958) and Denghai 605 (DH605) were used as experimental materials in a field experiment running from 2013 to 2015. Shading treatments were applied over different growth stages: from the tassel stage (VT) to physiological maturity (R6) (S1), from the six-leaf stage (V6) to VT (S2), and from emergence stage (VE) to R6 (S3). The effects of shading on plant photosynthesis, photosynthate accumulation and distribution, and yield were evaluated in comparison to ambient sunlight. Shading significantly decreased the leaf area, SPAD value, net photosynthetic rate, dry matter accumulation, and grain yield. During the 3-year experimental period, grain yields of ZD958 and DH605 were reduced by 83.4%, 34.2%, 53.1% and 79.3%, 24.2%, 57.6% as compared to the CK by treatments S3, S2, and S1, respectively. ^13^CO_2_ stable isotope tracing revealed that shading differentially affected the photosynthate transfer rate in different stages; photosynthates were transferred from top to bottom plant parts, in the order control > S2 > S1 > S3. We conclude that shading clearly disrupted photosynthate metabolism, and reduced the photosynthate accumulation in the grain, resulting in a yield reduction.

## Introduction

Light provides energy for the generation of plant assimilatory power and acts as a signal for photomorphogenesis (Kumar et al., 2016). Sufficient light is important for high and steady yields, especially in maize (*Zea mays* L.), which is a typical C4 plant. The high productivity of C4 plants is closely related to the differentiation and development of parenchyma, thus, C4 plants are very sensitive to light restriction (Ubierna et al., 2011; Chandra and Howard, 2014). CO_2_ has a high anti-diffusion effect in crops only under high light intensity, thereby enhancing CO_2_ fixation. Changes in light duration and intensity, both of which affect the structure and function of leaf mesophyll cells and vascular bundle sheath cells, also impact the synergistic effect of the C4 and C3 cycles (Evans et al., 2008; Tazoe et al., 2008; Kramer and Evans, 2011). In recent years, the low efficiency of C4 photosynthesis under low light conditions has been a topic of concern (Ubierna et al., 2011; Bellasio and Griffiths, 2014a).

In the North China Plain, under the influence of climatic and environmental conditions (e.g., wet weather, plant density, and altitude), summer maize is often subjected to low-light stress or self-shading in the later stages of growth. Increases in atmospheric aerosol content reduced the sunshine duration and solar radiation in the past 50 years, and the average annual temperature and frequency of extreme weather events are rising currently (Ren et al., 2005). There are more problems in maize production with the changes in climate, including shading, lodging and so on, which have a negative effect on national food security. For example, in the Huanghuaihai region, the rainy weather frequently occurs during the summer maize growing season (June–September), and can result in a 3–6% decrease in China’s total maize grain yield (Cui et al., 2013a). Previous studies indicated that shading decreased carbon fixation and canopy net photosynthetic rate (Hashemi-Dezfouli and Herbert, 1992; Kromdijk et al., 2008; Bellasio and Griffiths, 2014b), and the changes in photosynthetic organs occurred, such as leaf tissue morphology (Kloss and Schütze, 2015), stomatal movement (Kaushal et al., 2015), functional leaf chloroplast morphology and ultrastructure (Sheue et al., 2015), and chlorophyll content. In short, the low-light stress has negative effects on photosynthesis and grain yield of summer maize.

Crop yield is mainly determined by dry matter production and accumulation, which is also limited by the harvest index (Hu, 1995). Dry matter accumulation after anthesis in high-yield maize accounts for more than 60% of the total dry matter mass, and the harvest index is more than 0.53 (Lian et al., 2003). Dry matter formation and distribution in vegetative organs such as stem, leaf, and sheath ultimately determine maize grain yield (Huang Z.H. et al., 2007). The carbon transfer rate depends on the positions of the stem and leaves, which act as carbon sources (Wei et al., 2009). The distribution of photosynthate in the organs varies with the translocation of the growth center. Photosynthates are distributed mainly in the leaves before the nine-leaf vegetative stage (V9), and in stems and leaves at later stages. Assimilates accumulated before reproductive stage R3 (silk stage) contributes the most to grain formation. The carbon in the grain is derived from both photosynthesis and redistribution of carbon from vegetative organs. As photosynthesis gradually weakens after R3, dry matter in the grain is mainly translocated from the stem and leaves in this stage (Liu W. et al., 2011). Promoting the redistribution of carbon during later growth stages allows coordinating carbon metabolism and nitrogen metabolism, which requires carbon skeletons for N sequestration, so as to achieve higher crop yield and quality (Zhang et al., 2016). Based on previous research (Ren et al., 2005; Cui et al., 2013a, 2015), shading decreased dry matter accumulation and grain yields of summer maize. Thus, we hypothesized that under different shading conditions, the distribution of the ^13^C-photosynthate to the grains was different and shading after anthesis had greatest effects on photosynthate distribution and translocation compared with shading occured before anthesis. We tested this hypothesis by designing a field experiment in which shading occurred at different growth stages: S1, tassel stage (VT) to physiological maturity (R6); S2, six-leaf stage (V6) to VT; and S3, emergence to R6 using ^13^CO_2_ stable isotope tracer to explore the effect of shading in different period on ^13^C-photosynthate accumulation and distribution.

## Materials and Methods

### Experimental Site

The field experiment was conducted at the Experimental Farm of Shandong Agricultural University (36°09′N, 117°09′E, 158 m *a.s.l.*) and the State Key Laboratory of Crop Biology, China in the summer maize growing seasons of 2013–2015. The region is characterized by a temperate continental monsoon climate with an average annual temperature of about 12.9°C. The mean total precipitation that occurred during the summer maize growth periods in 2014 and 2015 was 240.0 and 282.6 mm, respectively. The soil type was brown soil, with a pH of 7.12 (Cambisol; FAO, 2003). The contents of organic matter, total N, total P, available N, rapidly available P, and rapidly available K in the 0–20 cm soil layer were 9.34 g kg^-1^, 0.76 g kg^-1^, 0.88 g kg^-1^, 80.61 mg kg^-1^, 37.19 mg kg^-1^, and 84.23 mg kg^-1^, respectively.

### Experimental Materials and Design

After harvesting of the winter wheat, two summer maize hybrids, Zhengdan 958 (ZD958) and Denghai 605 (DH605), which are the most popular varieties in China, were used as experimental materials. Maize was sown on June 15 in both years at a plant density of 67,500 plants per hectare. Four treatments were arranged in a split-plot randomized complete-block design with three replicates: ambient sunlight was used as a control (CK) and shading (40% of ambient light intensity) was applied during the following three growth periods: S1, tassel stage (VT) to physiological maturity (R6); S2, six-leaf stage (V6) to VT; and S3, emergence to R6. Each experimental plot was 27 m^2^ (3 × 9 m) in size and consisted of five rows of maize spaced 0.6 m apart. Shade cloth (Hongda Shade Cloth Company, Shouguang City, China) and scaffold formed a shed. A distance of 2 m between the shade cloth and the top of the maize canopy was maintained to keep the microclimate under the cloth consistent with that of the CK.

Fertilizers were applied at 240 kg N ha^-1^, 120 kg P_2_O_5_ ha^-1^, and 200 kg K_2_O ha^-1^ as urea (46% N), calcium dihydrogen phosphate (17% P_2_O_5_), and muriate of potash (60% K_2_O). Nitrogen fertilizer was sidedressed at V6 and 12-leaf stage (V12) at a ratio of 2:3, while P and K fertilizers were sidedressed at V6. Disease, weeds, and pests were well controlled in each treatment. Atrazine and acetochlor were surface applied before maize germination to control weeds, and phoxim was applied to control corn borer at V12.

### Sampling and Measurement

#### Field Microclimate

Irradiance was measured with a CI-110 plant canopy digital image analyzer (CID Company, Camas, WA, United States) placed 30 cm above the canopy. Canopy CO_2_ concentration and relative humidity were measured with a GXH-305 portable infrared CO_2_ instrument (Beijing Analytical Instrument Company, Beijing, China) and canopy air temperature with a thermometer at mid-plant height before VT and at ear height thereafter. Wind speed was determined with an AR816 anemometer (Huier Analytical Instrument Company, Hangzhou, China). Soil temperatures were determined with a geothermometer in the upper 0–5 cm of the soil (Ren et al., 2005; Cui et al., 2015). All parameters were measured at the center of each plot daily at 11:00 am for 7 days at VT. Means of three replications were calculated (**Table 9**).

#### Net Photosynthetic Rate (*Pn*)

Photosynthetic rates in ear leaves were measured at the middle of the uppermost and fully expanded leaves between 10:00 and 12:00 at V6, V12, VT, silk stage (R3), and R6 using a portable infrared gas analyzer (CIRAS II; PP Systems, Hansatech, United Kingdom). Five plants per treatment were randomly selected for measurements. Measurement conditions were kept consistent, the chamber was equipped with a red/blue LED light source. The PAR of CK was set at 1600 μmol m^-2^ s^-1^. The PAR of S was set at 500 μmol m^-2^ s^-1^.

#### Leaf Area Index (*LAI*)

Leaf length (L) and maximum leaf width (W) were measured in 15 representative plants per plot at V6, V12, VT, R3, and R6, and leaf area and LAI were calculated according to the method of Montgomery (1911).

$$\begin{array}{c} \text{Leaf}\;\text{area}\;\text{=}\;\text{L}\;\times \;\text{W}\;\times \;\text{0}.\text{75}\\ \text{LAI}\;\text{=}\;\frac{\left(\text{leaf}\;\text{area}\;\text{per}\;\text{plant}\;\times \;\text{plant}\;\text{number}\;\text{per}\;\text{plot}\right)}{\text{plot}\;\text{area}} \end{array}$$

#### Chlorophyll SPAD Value

The chlorophyll SPAD value was measured at V6, V12, VT, R3, and R6 in 10 randomly selected plants per treatment using a portable chlorophyll meter (SPAD-502, Soil-plant Analysis Development Section, Minolta Camera Co., Osaka, Japan).

#### Dry Matter Accumulation

Five representative plants were collected at V6, V12, VT, R3, and R6, and separated into leaves and stems (including stem, sheath, tassel, and ear-stalk) for V6, V12, and VT samples, and into leaves, stems, cob, and grain for R3 and R6 samples. The samples were oven-dried to constant weight at 80°C in a force-draft oven (DHG-9420A; Bilon Instruments, Shanghai, China) and then weighed.

#### ^13^C-Photosynthate Accumulation and Distribution

Ten representative plants in each plot were selected at V12. And the ear leaf of each selected plant was encased in a 0.1-mm-thick Mylar plastic bag, which permits sunlight into the bag at levels up to 95% of natural intensity. Bags were sealed at the base with Sellotape and injected with 50 mL ^13^CO_2_. After photosynthesis was allowed to proceed for 60 min, the ^13^CO_2_ in each bag was extracted through a KOH washer to absorb the remaining ^13^CO_2_, and the plastic bag was removed. This experiment was conducted on clear days between 9:00 AM and 11:00 AM.

The ear leaf and the leaf above and below this leaf were defined as the middle leaf, the leaves above the middle leaf were defined as the top leaf, and the leaves below the middle leaf were defined as the bottom leaf; the ear internode and the internodes immediately above and below were defined as the middle stem, the part above the middle stem was defined as top stem, and the part below the middle stem was defined as bottom stem.

Three plants of each treatment labeled with^13^C were harvested close to the ground at VT and R6 and were separated into top leaf, middle leaf, bottom leaf, top stem, middle stem, and bottom stem at VT, and into top leaf, middle leaf, bottom leaf, top stem, middle stem, and bottom stem, cob, and grain at R6. The samples were placed in paper bags, deactivated at 105°C for 30 min, dried to constant weight at 80°C, and weighed to record dry matter (g plant^-1^). All samples were ground into powder and passed through a 200-mesh sieve. ^13^C enrichment in 4-mg plant samples was determined by using an isotope 100 mass spectrometer (Isoprime, Manchester, United Kingdom), and the ^13^C allocation rate was calculated using the following equations (Wei et al., 2009):

^13^Cabundance :

$${\text{F}}_{\text{i}}\;(\%)\;=\;\frac{(\delta^{13}\text{C}\;+\;1000)\;\times \;{\text{R}}_{\text{PBD}}}{\left[(\delta^{13}\text{C}\;+\;1000)\;\times \;{\text{R}}_{\text{PBD}}\;+\;1000\right]}\;\times \;100$$

R_PBD_ (Carbon isotope ratio) = 0.0112372

Carbon content of each organ: C_i_ = total organ mass (g) × total carbon content (%) ^13^C (mg) into each component:

$${}^{13}{\text{C}}_{\text{i}}\;=\;\frac{{\text{C}}_{\text{i}}\;\times \;({\text{F}}_{\text{i}}\;-\;{\text{F}}_{\text{nl}})}{100}\;\times \;1000,$$

where Ci is the carbon content (g) contained in each component; nl is not labeled.

Net ^13^C assimilation by maize plants at the end of labeling was calculated by summing the ^13^C in each component. The percentage distribution of ^13^C into each component was calculated as:

$${}^{13}{\text{C}}_{\text{i}}\;(\%)\;=\;\frac{{}^{13}{\text{C}}_{\text{i}}}{{}^{13}{\text{C}}_{\text{net}\;\text{assimilation}}}\;\times \;100\%$$

#### Grain Yield, Yield Components, and Harvest Index

Thirty ears from the middle three rows of each plot were harvested at R6 using a continuous sampling method and were used to determine yield and yield components (standard moisture content is 14%). The harvest index was calculated by dividing the grain weight (standard moisture content is 14%) by the aboveground dry matter weight at R6.

### Statistical Analysis

The data were subjected to three-way analysis of variance (ANOVA). Growing season, blocks, and block interactions were included as random effects. Shading treatment and hybrids were included as fixed effects. In case of significant treatment effects, comparison of means was performed by means of LSD at a significance level of 0.05. LSD was used to compare adjacent means arranged in order of magnitude. ANOVA and the LSD test were conducted using the SPSS17.0 software program (Ver. 17.0, SPSS, Chicago, IL, United States). Figures were produced with Sigma Plot 12.5.

## Results

### Grain Yield, Yield Components, and Harvest Index

Grain yield, yield components, and harvest index for the two hybrids in 2013 to 2015 are shown in **Table 1**. There was no significant year × hybrid × treatment interaction effect on grain yield. Grain yields decreased significantly with respect to the CK after shading. In 2015, the grain yields of ZD958 and DH605 in S3, S2, and S1 were 83.2%, 41.4%, and 47.8%, and 74.2%, 33.4%, and 48.6% significantly lower than that of CK, respectively. Thus, the grain yield in S3 was the lowest, and that in S2 the highest. Shading significantly affected yield components: grains per ear and ear number of the two hybrids were reduced under shading as compared to CK, resulting in lower yield. In addition, the harvest indexes of the two hybrids were significantly decreased in S3 and S1 under shading. There were no interactions among year, hybrid and treatment on grain yield and its components. Three-year results are consistent.

**Table 1:** Grain yield and yield components of summer maize under different light treatments from 2013 to 2015.

Year	Hybrid	Treatment	Yield (kg ha^-1^)	1000-grain weight (g)	Grains per ear	Ear number (ears ha^-1^)	Harvest index
2013	ZD958	S3	2324d	278c	171d	49633d	0.25
		S2	8101b	310b	459b	57040b	0.48
		S1	5539c	279c	368c	54077c	0.44
		CK	11541a	351a	530a	62117a	0.45
	DH605	S3	2809d	229b	156d	54829c	0.32
		S2	9754b	347a	478b	58892b	0.47
		S1	4485c	315 b	253c	56300c	0.45
		CK	12554a	362a	545a	63593a	0.55
2014	ZD958	S3	1518d	203d	165d	45188d	0.18
		S2	8094b	277b	478b	61115b	0.46
		S1	4797c	250c	356c	54077c	0.45
		CK	11819a	334a	555a	63671a	0.56
	DH605	S3	1527d	216d	162d	43706d	0.20
		S2	9064b	304b	493b	60374b	0.69
		S1	4361c	290c	282c	53336c	0.45
		CK	10886a	320a	520a	653373a	0.57
2015	ZD958	S3	1802d	243d	190d	392593d	0.15
		S2	6285b	322b	385b	506173c	0.39
		S1	5593c	280c	359c	55803b	0.41
		CK	10723a	325a	568a	58129a	0.48
	DH605	S3	3056d	311d	260d	378605d	0.30
		S2	7888b	314b	450b	558645c	0.57
		S1	6087c	313c	327c	595065b	0.46
		CK	11850a	316a	568a	65965a	0.55
	ANOVA						
	Year(Y)		NS	NS	^∗^	NS	NS
	Hybrid(H)		^∗^	^∗^	^∗∗^	NS	^∗∗^
	Treatment(T)		^∗∗^	^∗∗^	^∗∗^	^∗∗^	^∗∗^
	Y × H		NS	NS	^∗^	NS	NS
	Y × T		^∗^	NS	NS	NS	NS
	H × T		^∗^	^∗^	^∗∗^	NS	^∗^
	Y × H × T		NS	NS	NS	NS	NS

*S3, shading from emergence stage to maturity stage (R6); S2, shading from six-leaf stage (V6) to tassel stage (VT); S1, shading from VT to R6; CK, natural lighting in the field. Values followed by a different small letter within a column are significantly different at 5% probability level. NS, not significant. ^∗^Significant at the 0.05 probability level. ^∗∗^Significant at the 0.01 probability level.*

**Table 2:** Correlation of yield, ear number, grains per ear and thousand-kernel weight from 2013 to 2015.

Correlations	Yield	Ear number	Grains per ear	TKW
Yield	1			
Ear number	0.177	1		
Grains per ear	0.984^∗∗^	0.187	1	
TKW	0.781^∗∗^	0.424	0.772^∗∗^	1

*TKW, thousand kernel weight. ^∗∗^Correlation is significant at the 0.01 level (two-tailed).*

### Net Photosynthetic Rate (*Pn*)

The *Pn* significantly changed after shading (**Table 3**). For all shading treatments, the *Pn* was lower than that of CK at the corresponding growth stages, and the greatest reduction was observed in S3. In 2015, the *Pn* of ZD958 and DH605 in S3, S2, and S1 was 59.2%, 31.7%, and 48.8%, and 46.7%, 14.0%, and 37.9% significantly lower than that of CK in R3, respectively. Thus, the largest decrease in *Pn* was observed in S3, followed by S1 and S2. Year and treatment had significant effect on *Pn* and there were no interactions among year, hybrid and treatment. The trend in 2014 was in accordance with that in 2015.

**Table 3:** Net photosynthetic rate in functional leaf of summer maize under different light treatments in 2014 and 2015 (μmol m^-2^ s^-1^).

Year	Hybrid	Treatment	V6	V12	VT	R3
2014	ZD958	S3	16b	17b	17b	16c
		S2	–	18b	19b	25b
		S1	–	–	–	16c
		CK	27a	30a	41a	28a
	DH605	S3	17b	17b	17b	16c
		S2	–	18b	17b	23b
		S1	–	–	–	17c
		CK	27a	33a	39a	28a
2015	ZD958	S3	13b	14b	18b	13d
		S2	–	15b	18b	20b
		S1	–	–	–	17c
		CK	27a	28a	36a	29a
	DH605	S3	16b	15b	18b	14d
		S2	–	18b	19b	22b
		S1	–	–	–	17c
		CK	27a	26a	34a	27a
	ANOVA					
	Year(Y)		^∗∗^	^∗∗^	^∗^	^∗∗^
	Hybrid(H)		NS	NS	NS	NS
	Treatment(T)		^∗∗^	^∗∗^	^∗∗^	^∗∗^
	Y × H		NS	NS	NS	NS
	Y × T		NS	^∗∗^	^∗∗^	^∗∗^
	H × T		^∗^	^∗∗^	NS	NS
	Y × H × T		NS	NS	NS	NS

*V6, six-leaf stage; V12, twelve-leaf stage; VT, tassel stage; R3, milking stage; S3, shading from emergence stage to maturity stage (R6); S2, shading from V6 to VT; S1, shading from VT to R6; CK, natural lighting in the field. Values fallowed by a different small letter within a column are significantly different at 5% probability level. NS, not significant. ^∗^Significant at the 0.05 probability level. ^∗∗^Significant at the 0.01 probability level.*

### Leaf Area Index (LAI)

The LAI of the two hybrids showed a single-peak curve, and the LAI under shading was lower than that in CK in the same period (**Table 4**). In 2015, the LAI of ZD958 in S3 was 23.9%, 37.5%, 25.5%, 34.7%, and 57.1% significantly lower than that of CK at each growth stage (V6, V12, VT, R3, and R6), respectively, and that of DH605 was 34.9%, 44.1%, 33.9%, 38.6%, and 36.3% significantly lower than that of CK, respectively. The LAI of ZD958 in S2 was 15.2%, 20.1%, 25.8%, and 9.0% lower than that of CK at each growth stage, respectively, and that of DH605 was 26.6%, 11.5%, 13.9%, and 6.8% lower than that of CK, respectively. Overall, the growth rate slowed down in S2 after the end of shading. The LAI of ZD958 and DH605 in S1 was 14.4%, 15.9%, and 12.1%, 11.1% lower than that of CK, respectively. The greatest LAI reduction occurred at S3. Year, hybrid (except at V6) and treatment had significant effect on LAI. The trend in 2014 was in accordance with that in 2015.

**Table 4:** Leaf area index of summer maize under different light treatments in 2014 and 2015.

Year	Hybrid	Treatment	V6	V12	VT	R3	R6
2014	ZD958	S3	0.6b	2.2c	2.8c	2.5c	1.2b
		S2	–	3.0b	3.2b	3.0b	1.7a
		S1	–	–	–	3.7a	1.6a
		CK	1.1a	3.9a	4.3a	3.8a	1.8a
	DH605	S3	0.4b	2.0c	2.8c	2.4b	1.5b
		S2	–	2.9b	3.2b	2.8b	1.9a
		S1	–	–	–	3.2a	1.9a
		CK	0.9a	3.6a	3.9a	3.6a	2.0a
2015	ZD958	S3	0.5b	2.4c	3.6c	2.8d	0.8c
		S2	–	3.3b	3.9b	3.2c	1.7a
		S1	–	–	–	3.7b	1.6b
		CK	0.7a	3.9a	4.8a	4.3a	1.9a
	DH605	S3	0.6b	2.1c	2.9c	2.4c	1.2c
		S2	–	2.8b	3.9b	3.4b	1.8b
		S1	–	–	–	3.4b	1.7b
		CK	0.9a	3.8a	4.5a	3.9a	1.9a
	ANOVA						
	Year(Y)		^∗^	^∗∗^	^∗∗^	^∗∗^	^∗∗^
	Hybrid(H)		NS	^∗∗^	^∗∗^	^∗∗^	^∗∗^
	Treatment(T)		^∗∗^	^∗∗^	^∗∗^	^∗∗^	^∗∗^
	Y × H		NS	^∗^	^∗∗^	NS	^∗^
	Y × T		^∗^	NS	^∗^	NS	^∗∗^
	H × T		NS	NS	^∗∗^	NS	^∗^
	Y × H × T		NS	^∗∗^	^∗∗^	^∗^	NS

*V6, six-leaf stage; V12, twelve-leaf stage; VT, tassel stage; R3, milking stage; R6, maturity stage; S3, shading from emergence stage to R6; S2, shading from V6 to VT; S1, shading from VT to R6; CK, natural lighting in the field. Values fallowed by a different small letter within a column are significantly different at 5% probability level. NS, not significant. ^∗^Significant at the 0.05 probability level. ^∗∗^Significant at the 0.01 probability level.*

### Chlorophyll SPAD Values

The different shading treatments significantly decreased the chlorophyll SPAD values with respect to the CK, with the most significant reduction noted in S3 (**Table 5**). Taking the results of 2015 as an example, the SPAD of ZD958 in S3 was 5.1%, 18.0%, 22.1%, 37.8%, and 43.7% significantly lower than that of CK at each growth stage, respectively, and that of DH605 was 10.1%, 18.2%, 20.9%, 38.3%, and 48.5% significantly lower than that of CK, respectively. The SPAD of ZD958 in S2 was 9.2%, 15.2%, 8.7%, and 32.5% significantly lower than that of CK at each growth stage, respectively, and that of DH605 was 8.3%, 13.7%, 14.7%, and 39.7% significantly lower than that of CK, respectively. This indicates that the growth rate slowed down in S2 after the end of shading. The SPAD of ZD958 and DH605 in S1 was 20.8%, 40.7% and 22.2%, 23.2% significantly lower than that of CK, respectively. The hybrid and treatment had significant effects on SPAD values. Results in 2014 showed a similar trend.

**Table 5:** SPAD value in functional leaves of summer maize under different light treatments in 2014 and 2015.

Year	Hybrid	Treatment	V6	V12	VT	R3	R6
2014	ZD958	S3	46b	46c	49c	34d	24c
		S2	–	52b	56b	53b	30b
		S1	–	–	–	49c	33b
		CK	54a	57a	59a	56a	47a
	DH605	S3	43b	45c	50c	33d	24c
		S2	–	55b	53b	57b	39b
		S1	–	–	–	54c	41b
		CK	55a	59a	64a	61a	50a
2015	ZD958	S3	48b	48c	47c	37d	24c
		S2	–	53b	51b	54b	29b
		S1	–	–	–	47c	25c
		CK	51a	58a	60a	60a	43a
	DH605	S3	49b	48c	50c	38d	28c
		S2	–	54b	55b	53b	32b
		S1	–	–	–	48c	41b
		CK	55a	59a	64a	62a	53a
	ANOVA						
	Year(Y)		NS	^∗^	NS	NS	^∗∗^
	Hybrid(H)		^∗^	^∗^	^∗∗^	^∗∗^	^∗∗^
	Treatment(T)		^∗∗^	^∗∗^	^∗∗^	^∗∗^	^∗∗^
	Y × H		^∗∗^	NS	^∗∗^	^∗∗^	^∗∗^
	Y × T		^∗∗^	NS	NS	^∗∗^	^∗∗^
	H × T		^∗^	NS	^∗^	^∗∗^	^∗∗^
	Y × H × T		NS	NS	^∗^	^∗∗^	^∗∗^

*V6, six-leaf stage; V12, twelve-leaf stage; VT, tassel stage; R3, milking stage; R6, maturity stage; S3, shading from emergence stage to R6; S2, shading from V6 to VT; S1, shading from VT to R6; CK, natural lighting in the field. Values fallowed by a different small letter within a column are significantly different at 5% probability level. NS, not significant. ^∗^Significant at the 0.05 probability level. ^∗∗^Significant at the 0.01 probability level.*

### Dry Matter

**FIGURE 1:**
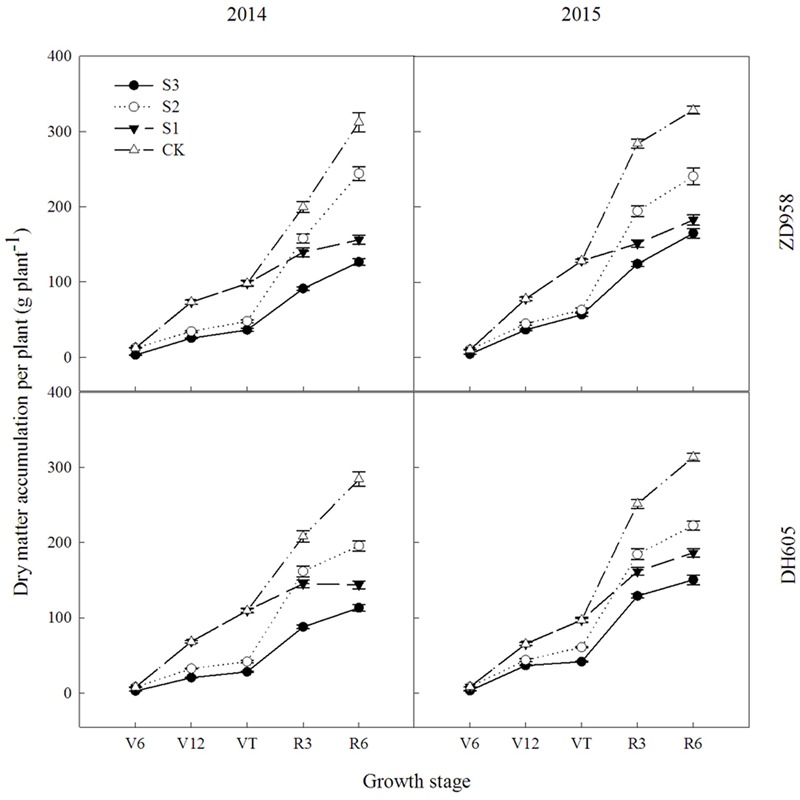
Dynamic changes in dry matter accumulation of summer maize under different treatments in 2014 and 2015 (g per plant). Means and standard errors based on three replicates are shown. S3, shading from emergence stage to maturity stage (R6); S2, shading from six-leaf stage (V6) to tassel stage (VT); S1, shading from VT to R6; CK, natural lighting in the field.

**FIGURE 2:**
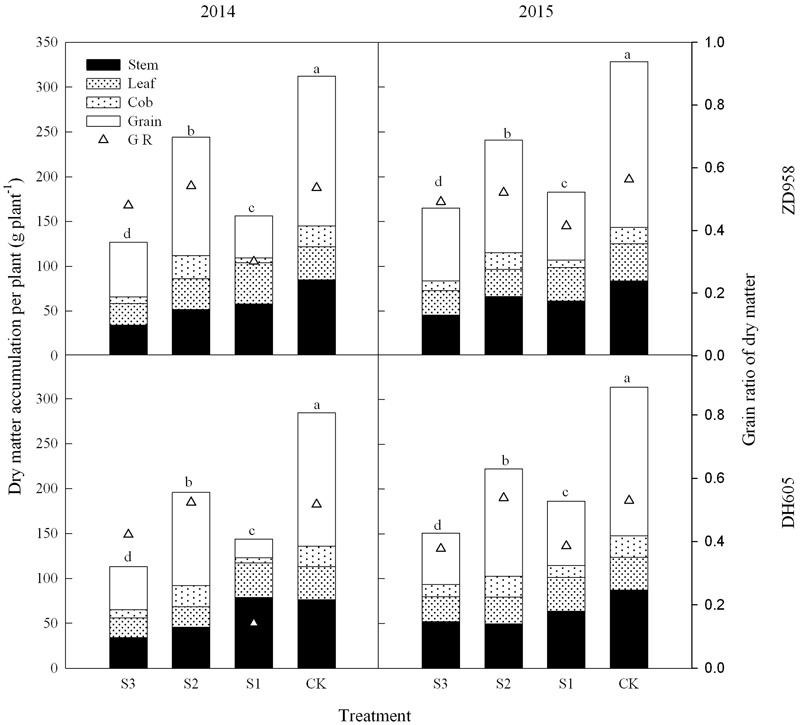
Histograms of dry matter accumulation and distribution of summer maize under different treatments at maturity stage (g per plant). The different small letters are significantly different at 5% probability level. S3, shading from emergence stage to maturity stage (R6); S2, shading from six-leaf stage (V6) to tassel stage (VT); S1, shading from VT to R6; CK, natural lighting in the field.

**Figure 1** shows that the dry matter accumulation in summer maize showed an “S-type” curve under the shading treatments at different stages. It increased slowly before VT, showed a steep increase at R3, and reached a maximum at R6. Taking the results of 2015 as an example, the dry matter accumulation of ZD958 in S3 significantly decreased by 56.5%, 52.9%, 55.9%, 56.3%, and 46.8% compared with CK at each growth stage, respectively, and that of DH605 significantly decreased by 60.7%, 43.7%, 57.0%, 48.6%, and 52.0% compared with CK, respectively. The dry matter accumulation of ZD958 in S2 significantly decreased by 42.4%, 50.9%, 31.5%, and 26.7% compared with CK at each growth stage, respectively, and that of DH605 significantly decreased by 32.8%, 37.2%, 26.6%, and 29.1% compared with CK, respectively. The dry matter accumulation of ZD958 and DH605 in S3 significantly decreased by 46.7% and 44.4%, and 35.7% and 45.8% compared with CK at each growth stage, respectively. Thus, shading not only reduces the amount of dry matter accumulated but also affects the proportion of dry matter accumulation and the distribution in the organs in different growth stages (**Figure 2**).

### Carbon Distribution

The largest effect of shading on the plant carbon content was observed in S3, while the smallest effect was found in S2 (**Table 6**). The stem carbon contents of ZD958 and DH605 showed similar trends: bottom stem > middle stem > top stem. The carbon distribution rates for ZD958 in top stem, middle stem, and bottom stem were 5.4%, 12.9%, 21.7% in S3; 3.1%, 10.0%, 16.6% in S2; 4.3%, 9.0%, 18.4% in S1; and 3.2%, 7.8%, 14.3% in CK, and those for DH605 were 5.9%, 13.4%, 20.4% in S3; 2.8%, 9.1%, 14.7% in S2; 4.6%, 14.5%, 17.2% in S1 and 3.3%, 9.0%, 11.7% in CK. The leaf carbon contents varied along the stem. The leaf carbon contents of ZD958 in S3, S1, and CK showed the same trend: bottom leaf > middle leaf > top leaf. The distribution rates for ZD958 in top leaf, middle leaf, and bottom leaf were 4.6%, 7.0%, 8.4% in S3; 2.9%, 4.0%, 3.5% in S2; 4.5%, 6.2%, 9.5% in S1; and3.2%, 3.5%, 5.3% in CK, respectively. Those for DH605 were 7.0%, 7.6%, 5.5% in S3; 3.0%, 4.0%, 3.6% in S2; 7.1%, 7.0%, 6.8% in S1 and 3.7%, 4.3%, 3.9% in CK, respectively. Grain carbon content showed the same trend in both hybrids: CK > S2 > S1 > S3.

**Table 6:** Plant carbon distribution of summer maize under different light treatments at maturity stage (g per plant).

Hybrid	Treatment	Stem			Leaf			Cob	Grain	Total
		Top	Middle	Bottom	Top	Middle	Bottom			
ZD958	S3	3.7b	9.0b	15.1c	3.2b	4.9b	5.9b	4.3c	23.6d	69.5d
	S2	3.7b	11.6a	19.3b	3.4b	4.6b	4.0c	6.6b	62.8b	116.0b
	S1	3.9b	8.1b	16.6c	4.1ab	5.6a	8.6a	3.5c	40.2c	90.5c
	CK	4.8a	11.8a	21.5a	4.8a	5.2ab	8.0a	8.9a	85.5a	150.5a
DH605	S3	4.1b	9.3b	14.1c	4.8c	5.3b	3.8c	5.8d	22.1d	69.2d
	S2	3.1c	10.0b	16.2b	3.3d	4.4c	4.0c	10.0b	59.1b	110.0b
	S1	4.6a	14.2a	16.9ab	7.0a	6.9a	6.6a	6.9c	35.1c	98.1c
	CK	5.0a	13.7a	17.9a	5.6b	6.6a	6.0b	12.0a	86.0a	152.8a

*S3, shading from emergence stage to maturity stage (R6); S2, shading from six-leaf stage (V6) to tassel stage (VT); S1, shading from VT to R6; CK, natural lighting in the field. Values fallowed by a different small letter within a column are significantly different at 5% probability level.*

### ^13^C-Photosynthate Accumulation and Distribution

The ^13^C isotope was concentrated in the middle and bottom parts of the stem and leaves in the flowering period, and started to be transferred to the grain after anthesis (**Table 7**). The middle stem of ZD958 and DH605 showed the highest transfer rates in S3, S1, and CK, with 13.0%, 66.7%, 51.6%, and 9.4%, 50.0%, 51.4%, respectively. In the stem of ZD958 in S2, ^13^C-photosynthate increased as compared to the CK. The bottom leaves of DH605 showed the highest transfer rates in S3, S2, S1, and CK, of 31.0%, 33.3%, 15.0%, and 22.5%, respectively. The top leaves of ZD958 had the highest rates in S3 and S1, with rates of 5.9%, and 19.2%, while the bottom leaves of ZD958 had the highest rates in S2 and CK, with values of 34.4%, and 12.8%. The grain transfer rates of ZD958 and DH605 showed similar trends: CK > S2 > S1 > S3.

**Table 7:** Distribution of ^13^C-photosynthate from functional leaf in maize plants.

Stage	Hybrid	Treatment	Top stem		Middle stem		Bottom stem		Top leaf		Middle leaf		Bottom leaf		Cob		Kernel	
			*g*	%	*g*	%	*g*	%	*g*	%	*g*	%	*g*	%	*g*	%	*g*	%
VT	ZD958	S3	1.5b	8.2a	5.4b	29.2b	5.7c	30.3b	1.7b	9.1a	2.2b	11.8a	2.1c	11.4c				
		S2	1.7b	7.9a	4.8c	22.6c	7.5b	35.3a	2.0b	9.2a	2.1b	10.0b	3.2b	15.1a				
		CK	2.7a	7.9a	12.6a	36.2a	9.4a	26.9c	2.6a	7.4b	2.8a	8.1c	4.7a	13.6b				
	DH605	S3	1.4b	7.8a	5.3b	29.7c	4.1c	22.7c	1.4b	7.7a	2.8b	15.7a	2.9b	16.4a				
		S2	1.7b	7.9a	5.4b	25.1c	7.1b	33.3a	1.8b	8.3a	2.4c	11.1b	3.0b	14.2b				
		CK	2.9a	7.7a	14.6a	38.1a	10.2a	26.8b	2.9a	7.6a	3.5a	9.3c	4.0a	10.5c				
R6	ZD958	S3	1.9b	5.3a	4.7b	13.0a	7.8c	21.7a	1.6b	4.6a	2.5b	7.0a	3.0b	8.4b	2.2c	6.1a	12.2d	33.9d
		S2	1.9b	3.2c	6.0a	10.0b	10.0b	16.7b	1.7b	2.9b	2.4b	4.0c	2.1c	3.5d	3.4b	5.7a	32.5b	54.1b
		S1	2.0b	4.3b	4.2b	8.9bc	8.6c	18.3b	2.1ab	4.4a	2.9a	6.1b	4.4a	9.4a	1.8c	3.9b	20.9c	44.6c
		CK	2.5a	3.2c	6.1a	7.8c	11.2a	14.3c	2.5a	3.1b	2.7ab	3.4d	4.1a	5.3c	4.6a	5.9a	44.4a	56.9a
	DH605	S3	2.1b	5.9a	4.8b	13.5a	7.3c	20.4a	2.5c	7.0a	2.7b	7.6a	2.0b	5.5b	3.0d	8.4ab	11.4d	31.8d
		S2	1.6c	2.8c	5.2b	9.2b	8.4b	14.8c	1.7d	3.0b	2.3c	4.0b	2.0b	3.6c	5.2b	9.1a	30.4b	53.5b
		S1	2.3ab	4.6b	7.3a	14.5a	8.7ab	17.1b	3.6a	7.1a	3.5a	7.0a	3.4a	6.7a	3.6c	7.1b	18.3c	35.9c
		CK	2.6a	3.2c	7.1a	9.0b	9.2a	11.6d	2.9b	3.7b	3.4a	4.3b	3.1a	3.9c	6.2a	7.8b	44.7a	56.4a

*S3, shading from emergence stage to maturity stage (R6); S2, shading from six-leaf stage (V6) to tassel stage (VT); S1, shading from VT to R6; CK, natural lighting in the field. Values fallowed by a different small letter within a column are significantly different at 5% probability level.*

## Discussion

### Effects of Shading on Field Microclimate and Fertility

No significant difference on microclimatic indexes, except light intensity, was observed. The same results were reported by Du et al. (2011). Previous studies suggested that under shading, when the plant obtains less energy, fertility is significantly delayed, and the degree of retardation is related to the degree (Ubierna et al., 2011), duration (Chandra and Howard, 2014), and period of shading (Evans et al., 2008). According previous researches, the sensitivity of maize to shading is different significantly. In this study, the greatest impact of shading on the fertility process of summer maize was observed in treatment S3 resulting from shading prevent pollen from scattering (**Table 10**).

### Effects of Shading on Photosynthetic Properties

Leaf photosynthetic performance and yield are closely related, and increasing the leaf photosynthetic performance is one of the key measures for obtaining high yields in summer maize (Liu et al., 2015; Ren et al., 2016). Theoretically, photosynthetic area, capacity, and time are important parameters determining photosynthetic performance. As leaves catch light energy, the sum of their sizes to a certain extent reflects the light interception and energy conversion capacity of the canopy (Li et al., 2010). The length of the functional period of maize leaves from flowering to silk ripening and the amount of dry matter accumulation after silking directly determine the yield (Tollenaar and Daynard, 1982; Sen et al., 2016). Previous studies have shown that under low-light stress, maize leaves lose chlorophyll and show early failure, resulting in a decrease in photosynthetic leaf area, and consequently, in yield loss (Jia et al., 2010; Cui et al., 2013b; Kong et al., 2012). Our results corroborated that the source such as photosynthetic leaf area and photosynthetic performance decreased under shading.

A previous study suggested that leaf chlorophyll content and photosynthetic rate in theory determine the potential grain yield of summer maize (Dong et al., 1997; Zhong et al., 2014). Higher rate and longer functional period of photosynthesis at the late growth stage benefit maize grain yield. In this study, it was found that after shading, the SPAD value and net photosynthetic rate decreased, photosynthetic function decayed rapidly, and dry matter quality and yield decreased. After the light was restored in S2, the photosynthetic function of the leaf was restored, but did not entirely remove the effects of shading, which remained significant, perhaps because shading might have damaged the leaf photoprotection mechanisms.

### Effects of Shading on Dry Matter Accumulation and Distribution

Photosynthesis produces more than 90% of the plant dry matter. Especially, the dry matter distribution and transport characteristics in the late growth stage determines the final yield in summer maize (Huang Z.X. et al., 2007; Chen et al., 2008). The movement of photosynthates from different plant parts to the reproductive organs is determined by the sink strength of the latter (Wei et al., 2009). Sink activity and carbon demand by the sink are considered the main drivers of carbon allocation in plants (Roland et al., 2015). Shading reduces carbon uptake and impairs photosynthetic performance in maize plants (Cui et al., 2013a). Our results showed that dry matter accumulation was consistently and significantly reduced under the shading regimens, which was in accordance with previous observations (Cui et al., 2015). Shading impaired sink strength including decreasing grains per ear and delaying development of endosperm cell (Gao et al., 2017), which decreased kernel number per ear and 1000-kernel weight. Before flowering, shading affected the reproductive growth of the plant, and dry matter accumulation decreased. After flowering, upon restoration of light in S2, plant growth was rapidly restored and dry matter accumulation increased. Shading in S2 affected the dry matter accumulation before VT, resulting in decreases in source and total dry matter. Shading in S1 affected grain filling, weakened the sink, and decreased grain number and grain weight, eventually leading to decreases in total dry matter accumulation and yield.

### Effects of Shading on ^13^C-Photosynthate Accumulation and Distribution

As dry matter accumulation and transportation are important factors determining maize yield, it is important to understand the contribution of dry matter from different parts to grain yield, to allow the development of improved summer maize varieties (Song and Tong, 2010). The dry matter accumulation pre-anthesis is mainly used for plant formation, such as the formation and growth of roots, stem, leaves, spikes, and other organs. The dry matter accumulated after anthesis is used mainly for grain formation; accordingly, high dry matter productivity after anthesis is one of the high-yield characteristics of summer maize (Liu et al., 2010; Zhang et al., 2011). Although the total ^13^C fixed was underestimated because the amount of ^13^C lost to the soil was not measured (Wei et al., 2009), assessing the amount of ^13^CO_2_ fixed in maize plants and translocated to each component provided an efficient way to assess the contribution of each part to the grain under shading stress. The ^13^C isotope tracer experiment showed that the distribution and transportation of photosynthate were different in each treatment. Shading significantly reduced the dry matter accumulation in each organ, and moreover, the proportion of dry matter allocated to the stem and leaves were increased, while that allocated to the grain was reduced. Photosynthates of top, middle, and bottom stem and leaves contributed to grain yield, which is consistent with the report of Li et al. (2012). In the early stages of maize grain sink formation, most of the ^13^C assimilates were distributed in the stalks, and then distributed to the leaves, and the distribution ratio in the stems was approximately 70% (**Table 7**). After flowering, ^13^C photosynthates in S3 were used for plant rather than grain formation, although the contribution to grain yield could not be ignored. After return to ambient light in S2, after anthesis, plant formation and grain enrichment took place simultaneously, and the transfer rates in stem, leaves, and other vegetative organs increased. However, dry matter accumulation in the stem, leaves, and “temporary” storage organs was low, and thus, grain could not get sufficient carbon from the vegetative organs and yield could not be restored to the normal level. Photosynthesis and thus, photosynthate, in S1 decreased after anthesis, and plants senesced prematurely. This had a negative effect on grain filling, reducing sink activity. The unbalanced source-to-sink ratio led to more ^13^C photosynthate in the stalk, thus decreasing yield (**Table 8**).

**Table 8:** Correlation of yield and distribution of ^13^C-assimilate from functional leaf of summer maize (%).

Correlations	Yield	Top stem	Middle stem	Bottom stem	Top leaf	Middle leaf	Bottom leaf	Cob	Kernel
Yield	1								
Top stem	-0.808*	1							
Middle stem	-0.717*	0.802*	1						
Bottom stem	-0.978**	0.848**	0.671	1					
Top leaf	-0.533	0.827*	0.859**	0.535	1				
Middle leaf	-0.799*	0.955**	0.855**	0.800*	0.888**	1			
Bottom leaf	-0.549	0.61	0.301	0.638	0.41	0.654	1		
Cob	0.131	-0.032	0.246	-0.235	0.176	-0.018	-0.626	1	
Kernel	0.859**	-0.958**	-0.897**	-0.862**	-0.864**	-0.979**	-0.611	-0.031	1

*^∗^Correlation is significant at the 0.05 level (two-tailed). ^∗∗^Correlation is significant at the 0.01 level (two-tailed).*

**Table 9:** Microclimate in experimental field under different light treatments from 2013 to 2015.

Year	Treatment	Air speed (m s^-1^)	Air temperature (°C)	Soil temperature (°C)	Relative humidity (%)	Light intensity (μmol m^-2^ s^-1^)	CO_2_ concentration (μmol mol^-1^)
2013	S	0.8a	29a	24a	51a	647b	334a
	CK	0.8a	31a	25a	51a	1592a	329a
2014	S	0.9a	26a	23a	47a	712b	327a
	CK	0.9a	25a	22a	52a	1675a	318a
2015	S	0.8a	28a	24a	53a	689b	329a
	CK	0.9a	30a	24a	52a	1681a	325a

*S, shading in the field; CK, natural lighting in the field. Values fallowed by a different small letter within a column are significantly different at 5% probability level.*

**Table 10:** Developmental progress of summer maize under different light treatments in 2014 and 2015 (M/D).

Year	Hybrid	Treatment	Sow	VE	V6	V12	VT	R3	R6
2014	ZD958	S3	6/17	6/22	7/19	8/6	8/13	9/13	10/16
		S2	6/17	6/22	7/14	8/3	8/12	9/6	10/7
		S1	6/17	6/22	7/14	7/29	8/8	9/7	10/8
		CK	6/17	6/22	7/14	7/29	8/8	9/3	10/5
	DH605	S3	6/17	6/22	7/19	8/6	8/13	9/13	10/16
		S2	6/17	6/22	7/14	8/3	8/12	9/6	10/7
		S1	6/17	6/22	7/14	7/29	8/9	9/7	10/8
		CK	6/17	6/22	7/14	7/29	8/9	9/4	10/5
2015	ZD958	S3	6/11	6/16	7/13	8/5	8/11	9/12	10/15
		S2	6/11	6/16	7/8	7/30	8/9	9/4	10/6
		S1	6/11	6/16	7/8	7/25	8/4	9/5	10/8
		CK	6/11	6/16	7/8	7/25	8/4	9/1	10/3
	DH605	S3	6/11	6/16	7/13	8/5	8/11	9/12	10/15
		S2	6/11	6/16	7/8	7/30	8/9	9/4	10/6
		S1	6/11	6/16	7/8	7/26	8/5	9/5	10/8
		CK	6/11	6/16	7/8	7/26	8/5	9/2	10/4

*VE, emergence stage; V6, six-leaf stage; V12, twelve-leaf stage; VT, tassel stage; R3, milking stage; R6, maturity stage; S3, shading from emergence stage to R6; S2, shading from V6 to VT; S1, shading from VT to R6; CK, natural lighting in the field.*

### Effects of Shading on Grain Yield and Yield Components

Appropriate light intensity and photoperiod are important for high and stable yields (Du et al., 2011; Liu Z.F. et al., 2011). Weak light in the later stages of maize growth results in smaller endosperm cells and lower grain weight at maturity and thus, directly affects yield (Li and Cui, 1989; Setter and Flannigan, 1989). Cui et al. (2015) pointed out that female spikelet differentiation is very sensitive to light; spike differentiation was retarded under shading, which decreased grains per ear and yield. The current study showed that shading reduced the maize grain yield, in the order S3 > S1 > S2, which was consistent with our previous findings (Li et al., 2005; Cui et al., 2013b). The correlations between yield and these parameters (**Table 2**) revealed that increasing the grains per ear and thousand-kernel weight with constant ear number might be an effective way to obtain high yield under shading.

## Conclusion

Shading significantly decreased the photosynthetic leaf area, SPAD value, net photosynthetic rate, and dry matter accumulation, while changing the distribution of dry matter in various organs and reducing the dry matter quality in the grain, thus lowering the grain yield. Therefore, this study suggests that the sowing date should be adjusted to avoid rainy weather in the late growing period, to ensure appropriate light conditions for the transport of photosynthates to safeguard yield. Reasonable fertilizer and water management should ensure that the photosynthates are efficiently transported to the grain. Thus, future research should focus on improving cultivation techniques, increasing photosynthetic efficiency, prolonging the photosynthetic function period, and promoting nutrient translocation to the grain under shading for harmonizing production and the environment.

## Author Contributions

JG carried out the measurements, data analysis, and drafted the manuscript. JZ designed the experiment. BZ, SD, PL, BR, and JZ made substantial contributions to conception, and critically revised the manuscript.

## Conflict of Interest Statement

The authors declare that the research was conducted in the absence of any commercial or financial relationships that could be construed as a potential conflict of interest. The reviewer EN and handling Editor declared their shared affiliation.
